# Cumulative Social Risk and Cardiovascular Disease Among Adults in South Korea: A Cross-Sectional Analysis of a Nationally Representative Sample

**DOI:** 10.5888/pcd17.190382

**Published:** 2020-05-28

**Authors:** Harold H. Lee, Augustine W. Kang, Hyunjoon Lee, Yoojin Cha, Don Operario

**Affiliations:** 1 Deparment of Social and Behavioral Sciences, Harvard T.H. Chan School of Public Health, Boston, Massachusetts; 2 Department of Behavioral and Social Sciences, Brown University School of Public Health, Providence, Rhode Island; 3 Department of Computer Science, Brown University, Providence, Rhode Island

## Abstract

**Introduction:**

The Framingham risk score (FRS) is widely used to predict cardiovascular disease (CVD), but it neglects to account for social risk factors. Our study examined whether use of a cumulative social risk score in addition to the FRS improves prediction of CVD among South Korean adults.

**Methods:**

We used nationally representative data on 19,147 adults aged 19 or older from the Korea National Health and Nutrition Examination Survey 2013–2016. We computed a cumulative social risk score (range, 0–3) based on 3 social risk factors: low household income, low level of education, and single-living status. CVD outcomes were stroke, myocardial infarction, and angina. Weighted logistic regression examined the associations between cumulative social risk, FRS, and CVD. McFadden pseudo-*R* ^2^ and area under receiver operating characteristic curve (AUC) assessed model performance. We conducted mediation analyses to quantify the association between cumulative social risk score and CVD outcomes that is not mediated by the FRS.

**Results:**

A unit increase in social risk was associated with 89.4% higher risk of stroke diagnosis, controlling for the FRS (*P* < .001). The FRS explained 8.0% of stroke diagnosis (*R* ^2^) with fair discrimination (AUC = 0.728), and adding the cumulative social risk score enhanced *R* ^2^ and AUC by 2.4% and 0.039. In the association between cumulative social risk and stroke, the proportion not mediated by the FRS was 65% (*P* < .001). We observed similar trends in myocardial infarction and angina, such that an increase in social risk was associated with increased relative risk of disease and improved disease diagnosis, and a large proportion of the association was not mediated by the FRS.

**Conclusion:**

Controlling for the FRS, cumulative social risks predicted stroke, myocardial infarction, and angina among adults in South Korea. Future research is needed to examine non-FRS mediators between cumulative social risk and CVD.

**Summary**

**What is already known on this topic?**

The Framingham risk score is a robust algorithm to predict cardiovascular disease (CVD) risk based on demographic and clinical factors among European Americans.

**What is added by this report?**

The Framingham risk score was predictive of stroke, myocardial infarction, and angina among a nationally representative sample of adults in South Korea. In addition, cumulative social risk was predictive of CVD incidence, independently of Framingham risk score.

**What are the implications for public health practice?**

The effect of cumulative social risk on CVD may not be fully mediated by poor health behaviors (eg, smoking) and cardiometabolic profile (eg, blood pressure, diabetes, cholesterol). Further investigation of nonbiobehavioral mediators between social factors and CVD is warranted.

**MEDSCAPE CME**

In support of improving patient care, this activity has been planned and implemented by Medscape, LLC and *Preventing Chronic Disease*. Medscape, LLC is jointly accredited by the Accreditation Council for Continuing Medical Education (ACCME), the Accreditation Council for Pharmacy Education (ACPE), and the American Nurses Credentialing Center (ANCC), to provide continuing education for the healthcare team.

Medscape, LLC designates this Journal-based CME activity for a maximum of 1.00 AMA PRA Category 1 Credit(s)™. Physicians should claim only the credit commensurate with the extent of their participation in the activity.

Successful completion of this CME activity, which includes participation in the evaluation component, enables the participant to earn up to 1.0 MOC points in the American Board of Internal Medicine’s (ABIM) Maintenance of Certification (MOC) program. Participants will earn MOC points equivalent to the amount of CME credits claimed for the activity. It is the CME activity provider’s responsibility to submit participant completion information to ACCME for the purpose of granting ABIM MOC credit.

**Release date: May 28, 2020; Expiration date: May 28, 2021**

**Learning Objectives**

Upon completion of this activity, participants will be able to:

1. Describe the contribution of CSRS to stroke risk beyond the FRS among South Korean adults, according to study results using nationally representative data from KNHANES from 2013 to 2016
2. Determine the contribution of CSRS to risk for myocardial infarction (MI) and angina beyond the FRS among South Korean adults, according to study results using nationally representative data from KNHANES from 2013 to 2016
3. Identify the clinical implications of contribution of CSRS to risk for CVD beyond the FRS among South Korean adults, according to study results using nationally representative data from KNHANES from 2013 to 2016

**EDITOR**

Ellen Taratus, MS, ELS

Editor

Preventing Chronic Disease

Disclosure: Ellen Taratus has disclosed no relevant financial relationships.

**CME AUTHOR**

Laurie Barclay, MD

Freelance writer and reviewer

Medscape, LLC

Disclosure: Laurie Barclay, MD, has disclosed no relevant financial relationships.

**AUTHORS**

Harold H. Lee, PhD

Department of Social and Behavioral Sciences

Harvard T.H. Chan School of Public Health

Boston, Massachusetts

Disclosure: Harold H. Lee, PhD, has disclosed no relevant financial relationships.

Augustine W. Kang, PhD

Department of Behavioral and Social Sciences

Brown University School of Public Health

Providence, Rhode Island

Disclosure: Augustine W. Kang, PhD, has disclosed no relevant financial relationships.

Hyunjoon Lee, BS

Data Science Initiative

Department of Behavioral and Social Sciences

Brown University School of Public Health

Providence, Rhode Island

Disclosure: Hyunjoon Lee, BS, has disclosed no relevant financial relationships.

Yoojin Cha, ScM

Department of Behavioral and Social Sciences

Brown University School of Public Health

Providence, Rhode Island

Disclosure: Yoojin Cha, ScM, has disclosed no relevant financial relationships.

Don Operario, PhD

Department of Behavioral and Social Sciences

Brown University School of Public Health

Providence, Rhode Island

Disclosure: Don Operario, PhD, has disclosed no relevant financial relationships.

## Introduction

Cardiovascular disease (CVD) has been the leading cause of death in the past 20 years (1). In 2016, ischemic heart disease and stroke accounted for a combined 26% (15.2 million) of global deaths (2). To curb the incidence of CVD, a comprehensive understanding of CVD etiology is needed.

Epidemiological and biomedical research has made progress in understanding CVD etiology and the mechanisms through which determinants manifest as CVD. Among the better-known studies is the Framingham Heart Study, an ongoing longitudinal cohort study. Many known determinants of CVD, such as smoking, cholesterol, blood pressure, and physical activity, were first reported by the Framingham Heart Study (3–5). The Framingham risk score (FRS) was developed to predict a person’s 10-year risk for CVD on the basis of demographic information, such as age and sex, and biobehavioral markers, such as cholesterol levels, smoking status, systolic blood pressure, and diabetes (6).

A limitation of the FRS is that it is based solely on individual-level factors. Hence, it does not sufficiently account for the role of social factors in determining CVD risk. For example, empirical data suggest that the FRS underestimates CVD mortality and morbidity among adults with low socioeconomic status in the United States (7) and Scotland (8). A wealth of evidence corroborates the relationship between socioeconomic factors (household income, education, occupation, marital status, social support) on health, with some research even suggesting a causal influence (9,10). Evidence on the social determinants of CVD has accumulated such that current scientific literature recommends including socioeconomic factors such as income, education, and social isolation in the FRS to enhance prediction of CVD (11,12). Associations between socioeconomic status and CVD risk persist even after controlling for age, sex, smoking, hypertension, diabetes, physical activity, diet, cholesterol, and body weight (13). In 2 recent studies, CVD researchers examined composite measures of cumulative social risk (14,15) by identifying indicators of social disadvantage that were consistently related to health outcomes, including household income, education, solitude (ie, whether one lives alone), and ethnicity (14,16). In a nationally representative sample of US adults, cumulative social risk factors were associated with increased CVD mortality (15). An important gap in this research, however, is that we do not know whether cumulative social risk predicts CVD independently of the individual-level biobehavioral factors measured in the FRS.

Most evidence on CVD risk is based on participants sampled in the Western Hemisphere, despite studies indicating that the prevalence of chronic disease is increasing rapidly across the world, in nations such as South Korea (17). The economic growth in South Korea since the 1960s (18) has been accompanied by increases in inequalities in income and education. Concerns are emerging about health disparities in overall mortality and the prevalence of noncommunicable diseases (19–21).

The objective of our study was to examine the effect of cumulative social risk and the FRS on CVD incidence among adults in South Korea, with the following 3 aims. First, we investigated the association between the FRS and CVD incidence to assess the utility of the FRS among adults in South Korea. Second, we assessed the association between a cumulative social risk score (based on low household income, low level of education, and single-living status) and CVD incidence. Finally, we examined the association between the cumulative social risk score and CVD incidence, controlling for the FRS.

## Methods

We analyzed nationally representative data from the 2013–2016 Korea National Health and Nutrition Examination Survey (KNHANES), conducted by the Korean Centers for Disease Control and Prevention (KCDC) and the Ministry of Health and Welfare. Data were collected from the publicly available KCDC website (http://knhanes.cdc.go.kr). A full description of KNHANES methodology is described elsewhere (22,23). In brief, KNHANES is a nationally representative, cross-sectional sample of the noninstitutionalized South Korean population. The KNHANES team used a multistage clustered probability design based on the administrative district, place of residence, and residential means (ie, apartment, other than apartment) to ensure recruitment of a representative sample. KNHANES team members visited each sampled household, where they conducted physical examinations for the health survey and face-to-face interviews for the nutrition survey. In KNHANES 2013–2015, 29,321 persons were asked to participate, and 22,948 (78.3%) agreed and responded to the survey. In KNHANES 2016, 10,806 were asked to participate, and 8,150 (75.4%) agreed and responded to the survey. From 31,188 people who participated in the one-time survey, 12,041 were excluded because of missing variables (Figure). Our study sample consisted of 19,147 participants.

**Figure:**
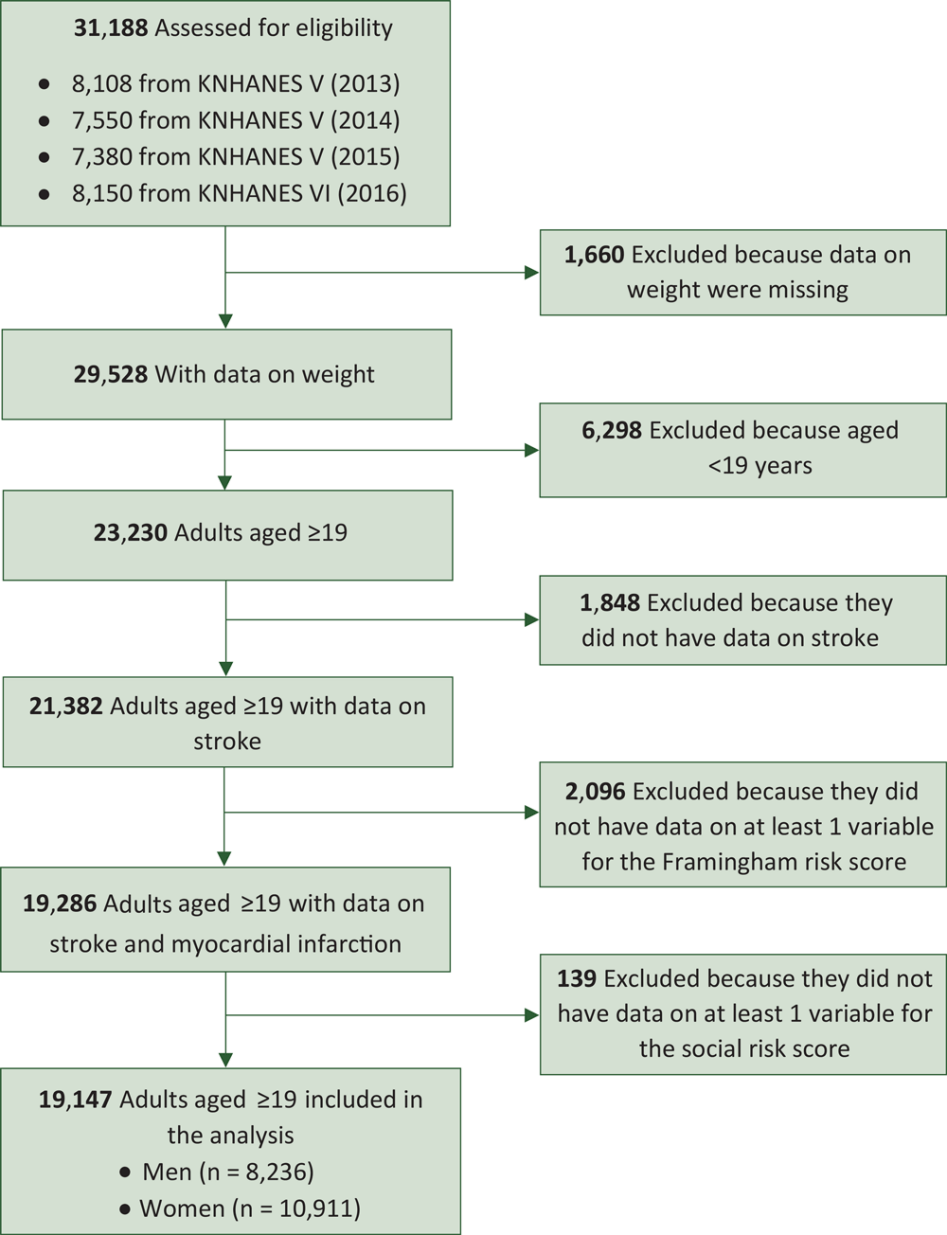
Potential participants included and excluded in the Korea National Health and Nutrition Examination Survey (KNHANES), 2013–2016.

Written informed consent was secured from all participants. The institutional review board of the KCDC approved this study, and the study protocol conformed to the ethical guidelines of the 1975 Declaration of Helsinki. The analysis was deemed exempt by the institutional review board of Brown University.

### Measures

We computed the FRS using the algorithm described by D’Agostino et al (6). Components of the risk score are age, total cholesterol, high-density lipoprotein cholesterol, smoking status, diabetes status, systolic blood pressure, and whether or not the participant was currently being treated for hypertension. Consistent with previous studies (14,15), we computed a cumulative social risk score (range, 0 to 3) by using 3 binary social risk factors: low household income (yes/no), low level of education (yes/no), and single-living status (yes/no). We did not include race or ethnicity in the cumulative social risk score because this information is not obtained by KNHANES, probably because South Korea is racially and ethnically homogeneous. KNHANES assessed household income by dividing monthly household income by the square root of the number of household members (adjusting for sex and each 5-year age stratum); participants were categorized into 4 quartiles of household income (upper, moderate, moderate-low, and low). We classified household income lower than the 50th percentile (ie, moderate-low and low) as low household income. KNHANES assessed education by using the question, “What is the highest qualification you obtained from school?” Response categories were college graduate, high school graduate, middle school graduate, and elementary school graduate. We classified middle-school graduate and elementary-school graduate as low level of education. KNHANES also assessed the number of people cohabitating per household by the question, “How many people are cohabiting with you?” Response categories ranged from 0 to 9 persons. Respondents who reported zero people were classified as single living. We examined 3 CVD outcomes: stroke, myocardial infarction, and angina. The occurrence of a lifetime CVD event was determined in the health interview survey in KNHANES. We classified participants who answered yes to the question, “Have you ever been diagnosed with (stroke, myocardial infarction, or angina) by a physician?” as persons with a previous CVD event.

### Analytic strategy

We conducted all analyses in R version 3.3.1 (24), and we applied weights by using the *svy* command. First, we conducted descriptive analyses to examine distributions for all key variables. Second, we used weighted logistic regressions to examine the effect of the cumulative social risk score, controlling for the FRS. McFadden pseudo-*R* ^2^ assessed the variability of CVD diagnoses explained by predictors in each model. Goodness-of-fit (*C* statistic) assessed the area under the receiver operating characteristic curve (AUC). The AUC can range from 0.50 to 1.00, with higher values indicating better predictive models. Although interpretation of AUC depends on context, a general guideline is that values above 0.80 indicate good models, between 0.70 and 0.80 fair models, and between 0.60 and 0.70 poor models (25). To adjust for multiple testing of 3 dependent variables (ie, stoke, myocardial infarction, and angina), we used a Bonferroni correction α of 0.016 (0.05/3). Finally, we used mediation modeling to quantify the proportion not mediated by the FRS in the association between cumulative social risk and CVD outcomes. We assessed the mediator (ie, FRS) through simple linear regression (ie, FRS regressed on cumulative social risk) and logistic regression models (ie, CVD regressed on cumulative social risk with FRS as covariate). We then combined these results to estimate direct and indirect effects, using product-of-coefficient methods (26).

## Results

The mean age of our KNHANES sample was 45.6 (standard deviation, 15.9) (Table 1). About three-quarters (76.9%) of the sample were married, and 8.0% lived alone. The sample was well educated, with 76.0% of the sample having graduated from high school; 24.0% had a low level of education. More than one-third (38.6%) had low household income. About half (49.2%) had at least 1 social risk factor. Of the study sample, 1.6% reported having a stroke, 0.7% a myocardial infarction, and 1.4% angina.

**Table 1:** Population-Weighted Characteristics of the Study Sample, Adults Aged ≥19 (n = 19,147) From the Korea National Health and Nutrition Examination Survey (KNHANES), 2013–2016

Characteristic	No. (Weighted %)
**Age, mean (SE), y**	45.6 (15.9)
**Female**	10,911 (50.3)
**Married**	16,102 (76.9)
**Social risk factors**	
Low household income^a^	8,223 (38.6)
Low level of education^b^	6,085 (24.0)
Single living^c^	1,852 (8.0)
**Framingham risk factors (except age and sex)**	
Total cholesterol, mean (SE), mg/dL	189.6 (35.7)
High-density lipoprotein cholesterol, mean (SE), mg/dL	51.2 (12.4)
Has ever smoked	7,076 (40.3)
Has diabetes	2,240 (9.5)
Systolic blood pressure, mean (SE), mm Hg	116.7 (15.8)
Receives treatment for hypertension	3,945 (15.5)
**Occurrence of a lifetime CVD event^d^**	
Stroke	420 (1.6)
Myocardial infarction	172 (0.7)
Angina	364 (1.4)
**Cumulative social risk score^e^**	
0	8,671 (50.8)
1	5,790 (31.0)
2	3,688 (15.1)
3	998 (3.1)

Abbreviation: CVD, cardiovascular disease.

a Participants were categorized into 4 quartiles of household income (upper, moderate, moderate-low, and low). Household income lower than the 50th percentile (ie, moderate-low and low) was categorized as low household income.

b KNHANES asked, “What is the highest qualification you obtained from school?” Response categories were college graduate, high school graduate, middle school graduate, and elementary school graduate. Middle school graduate and elementary school graduate were classified as low level of education.

c KNHANES asked, “How many people are cohabiting with you?” Response categories ranged from 0 to 9 persons. Respondents who reported zero people were classified as single living.

d KNHANES asked, “Have you ever been diagnosed with [stroke, myocardial infarction, or angina] by a physician?” Respondents who answered yes were categorized as having a lifetime occurrence.

e Possible range, 0–3: 0, no social risk factors reported; 1, 1 social risk factor reported; 2, 2 social risk factors reported; 3, 3 social risk factors reported.

A 1-unit increase in the cumulative social risk score was associated with 89.4% higher risk of having a stroke, controlling for the FRS (*P* < .001) (Table 2). The FRS alone explained 8.0% of the variance of a stroke diagnosis (*R* ^2^), with fair discrimination (AUC = 0.728). For this model, adding the cumulative social risk score improved *R* ^2^ by 2.4%, and the AUC was increased by 0.039.The shared contribution of the FRS and cumulative social risk score was 5.2%, with a unique contribution of 2.8% from the FRS and a unique contribution of 2.4% from the cumulative social risk score, altogether predicting 10.4% variation of stroke diagnosis. In the association between cumulative social risk score and stroke, the proportion mediated by the FRS was 35% (95% CI, 26%–45%, *P* < .001).

**Table 2:** Weighted Logistic Regression of Cardiovascular Disease on Framingham Risk Score^a^ and Cumulative Social Risk^b^, Adults Aged ≥19 (n = 19,147) From the Korea National Health and Nutrition Examination Survey, 2013–2016^c^

Cardiovascular Disease	Unadjusted	Adjusted^d^
**Stroke **		
Framingham risk score	1.81 (1.70–1.95) [<.001]	1.42 (1.31–1.53) [<.001]
Cumulative social risk score	2.53 (2.25–2.84) [<.001]	1.89 (1.64–2.17) [<.001]
**Myocardial infarction**		
Framingham risk score	1.66 (1.51–1.83) [<.001]	1.37 (1.22–1.55) [<.001]
Cumulative social risk score	2.11 (1.79–2.49) [<.001]	1.63 (1.31–2.01) [.002]
**Angina**		
Framingham risk score	1.88 (1.72–2.06) [<.001]	1.56 (1.41–1.73) [<.001]
Cumulative social risk score	2.35 (2.08–2.66) [<.001]	1.64 (1.42–1.89) [<.001]

a Computed by using the algorithm described by D’Agostino et al (6). Components of the risk score are age, total cholesterol, high-density lipoprotein cholesterol, smoking status, diabetes status, systolic blood pressure, and whether or not the participant was treated for hypertension.

b Consistent with previous studies (14,15), we computed a cumulative social risk score (range, 0 to 3) by using 3 binary social risk factors: low household income (yes/no), low level of education (yes/no), and single-living status (yes/no).

c All values are relative risk (95% confidence interval) [*P* value].

d In the adjusted model, each cardiovascular disease was regressed on the Framingham risk score and cumulative social risk.

A 1-unit increase in the cumulative social risk score was associated with 62.7% higher risk of having a myocardial infarction, controlling for the FRS (*P* < .001). The FRS alone explained 5.2% of the variance of a stroke diagnosis (*R* ^2^), with poor discrimination (AUC = 0.674). For this model, adding the cumulative social risk score improved *R* ^2^ by 1.1%, and the AUC was increased by 0.043, resulting in fair discrimination (AUC = 0.713). The shared contribution of the FRS and the cumulative social risk score was 3.1%, with a unique contribution of 2.1% from the FRS and a unique contribution of 1.1% from the cumulative social risk score, altogether predicting 6.3% variation of stroke diagnosis. In the association between the cumulative social risk score and myocardial infarction, the proportion mediated by the FRS was 40% (95% CI, 24%–62%, *P* < .001).

A 1-unit increase in the cumulative social risk score was associated with 63.6% higher risk of having angina, controlling for the FRS (*P* < .001). The FRS alone explained 6.3% of the variance of an angina diagnosis (*R* ^2^), with fair discrimination (AUC = 0.734). For this model, adding the cumulative social risk score improved *R* ^2^ by 3.7%, and the AUC was increased by 0.023. The shared contribution of the FRS and cumulative social risk score was 4.9%, with a unique contribution of 3.7% from the FRS and a unique contribution of 1.4% from the cumulative social risk score, altogether predicting 10.0% variation of angina diagnosis. In the association between the cumulative social risk score and angina, the proportion mediated by the FRS was 45% (95% CI, 35%–58%, *P* < .001).

## Discussion

Cumulative social risk predicted 3 CVD outcomes (stroke, myocardial infarction, and angina) after controlling for the FRS and taking into consideration multiple testing. Our study demonstrated improved model prediction, which suggests that cumulative social risk may bear clinical significance.

To our knowledge, ours is the first study to examine cumulative social risk in relation to CVD independently of the FRS and the first to examine this relationship in a nationally representative sample of South Korean adults. Although no previous studies examined the relation between cumulative social risk and CVD controlling for the FRS, findings from our study are consistent with findings from previous studies that examined the association between socioeconomic status — a factor that largely overlaps with cumulative social risk — and CVD, controlling for the FRS. One study investigated the association between socioeconomic status (measured as <12 years of education or low income) and coronary heart disease, controlling for the FRS (7). Adding the socioeconomic status variable to a model with the FRS improved calibration, with predicted risk estimates of 3.1% for those with higher socioeconomic status and 5.2% for those lower socioeconomic status; inclusion of socioeconomic status in the model resulted in upgrading risk classification for 15.1% of participants with low socioeconomic status. Similarly, in a prospective study of 12,304 men and women in western Scotland, the FRS underestimated CVD among manual workers, compared with nonmanual workers, and among people from deprived areas, compared with people from affluent areas (8).

In our study, cumulative social risk predicted CVD among adults in South Korea. Future comparative research is needed to examine whether the effect of cumulative social risk on CVD is stronger in South Korea than in the Western Hemisphere (eg, United States) or whether the effect is similar across contexts. Investigating the presence and size of this effect across contexts has public health relevance because such studies can inform whether interventions or policies to reduce CVD need to be culturally tailored according to geographical or cultural contexts. Although empirical studies appear to favor the idea that the link between cumulative social risk and CVD is a generalizable trend (14–16), research is needed to ascertain whether the findings from our study are replicable in other settings and to identify the mechanisms by which cumulative social risk leads to CVD.

In the association between cumulative social risk and CVD outcomes, the proportion mediated by the FRS was 35% to 45%. Although this association corroborates the importance of intervening at the components of the FRS (eg, smoking, total cholesterol, blood pressure), it also highlights that a substantial proportion (ie, ~55%–65%) is not mediated by the FRS. Put differently, cumulative social risk may hypothetically influence CVD through nonbiobehavioral factors assessed in the FRS, such as increased levels of psychological stress (27) or decreased levels of optimism (28). Most empirical epidemiological studies that examined cumulative social risk on somatic health (eg, CVD, cancer, mortality) highlighted the possibility that cumulative social risk plays a key role in life-course epidemiology (eg, parenting and infant development, early childhood, transition to adulthood) (29). Taken together, research that examines the nonbehavioral pathways and developmental processes connecting cumulative social risk and CVD outcomes is warranted.

Our study has several strengths. First, it used a nationally representative sample of adults in South Korea. It had 3 CVD measures and found effects for all 3 outcomes. However, our study also had several limitations. KNHANES has a cross-sectional design, and, therefore, we cannot infer causality. Reverse causality is theoretically possible, whereby incidence of CVD (ie, unhealthiness) may cause low income, low levels of education, or solitude, although little empirical data exist to support these causal pathways. Because survey data were collected through interviews in participants’ households, responses might have been affected by self-report bias. Also, although the FRS encompasses some health behaviors (eg, smoking) and their downstream factors (eg, hypertension, diabetes), in the mediation analyses, we did not control for health behaviors that could lie in the pathway between cumulative social risk score and CVD, such as physical activity and diet (30). Although KNHANES asked participants whether CVD had been diagnosed by a physician, these data were self-reported. In addition, most types of angina may be classified as a CVD outcome, but our data did not differentiate between CVD-related angina and non-CVD–related angina. Angina may not be related to CVD if a person has no documented CVD or no risk factors for CVD.

The FRS predicted stroke, myocardial infarction, and angina in a nationally representative sample of South Korean adults. Moreover, a cumulative social risk measure that incorporated income, education, and single living predicted CVD independently of the FRS, suggesting that the potential effect of cumulative social risk on CVD may not be fully mediated by biological and behavioral markers assessed by the FRS (eg, smoking, blood pressure, diabetes, cholesterol). Future investigation is warranted to understand the nonbiobehavioral mediators (ie, factors not included in the FRS) between social risk and CVD.

## Post-Test Information

To obtain credit, you should first read the journal article. After reading the article, you should be able to answer the following, related, multiple-choice questions. To complete the questions (with a minimum 75% passing score) and earn continuing medical education (CME) credit, please go to http://www.medscape.org/journal/pcd. Credit cannot be obtained for tests completed on paper, although you may use the worksheet below to keep a record of your answers.

You must be a registered user on http://www.medscape.org. If you are not registered on http://www.medscape.org, please click on the “Register” link on the right hand side of the website.

Only one answer is correct for each question. Once you successfully answer all post-test questions, you will be able to view and/or print your certificate. For questions regarding this activity, contact the accredited provider, CME@medscape.net. For technical assistance, contact CME@medscape.net. American Medical Association’s Physician’s Recognition Award (AMA PRA) credits are accepted in the US as evidence of participation in CME activities. For further information on this award, please go to https://www.ama-assn.org. The AMA has determined that physicians not licensed in the US who participate in this CME activity are eligible for AMA PRA Category 1 Credits™. Through agreements that the AMA has made with agencies in some countries, AMA PRA credit may be acceptable as evidence of participation in CME activities. If you are not licensed in the US, please complete the questions online, print the AMA PRA CME credit certificate, and present it to your national medical association for review.

## Post-Test Questions

### Study Title: Cumulative Social Risk and Cardiovascular Disease Among Adults in South Korea: A Cross-Sectional Analysis of a Nationally Representative Sample

#### CME Questions

1. You are advising a large cardiology practice regarding determination of CVD risk. According to study results using nationally representative data from KNHANES from 2013 to 2016 by Lee and colleagues, which of the following statements about the contribution of CSR to stroke risk beyond the FRS among South Korean adults is correct?
  - After controlling for FRS, a 1-unit increase in CSRS was associated with an 89.4% higher risk for stroke diagnosis (*P* < .001)
  - Adding CSRS to FRS did not improve risk prediction by McFadden pseudo-*R* ^2^ and area under the receiver-operating characteristic curve (AUC)
  - FRS explained 48% variance of stroke diagnosis (*R* ^2^) with good discrimination
  - In the association between CSRS and stroke, the proportion not mediated by FRS was 25%
2. According to study results using nationally representative data from KNHANES from 2013 to 2016 by Lee and colleagues, which of the following statements about risk for MI and angina beyond the FRS among South Korean adults is correct?
  - After controlling for FRS, CSRS was not associated with risk for MI
  - The FRS alone explained 25.2% of the variance in stroke diagnosis (*R* ^2^), with fair discrimination according to AUC
  - After controlling for FRS, a 1-unit increase in CSRS was associated with a 63.6% higher risk for angina (*P* < .001)
  - Adding CSRS to FRS did not improve risk prediction for angina according to *R* ^2^ or AUC
3. According to study results using nationally representative data from KNHANES from 2013 to 2016 by Lee and colleagues, which of the following statements about the clinical implications of contribution of CSRS to risk for CVD beyond the FRS among South Korean adults is correct?
  - The study identified non-FRS mediators between CSRS and CVD
  - Findings from this study are inconsistent with prior studies of the association between socioeconomic status and CVD, after controlling for FRS
  - The potential effect of CSRS appears to be fully mediated by biological and behavioral markers assessed in FRS (eg, smoking, blood pressure, diabetes, and cholesterol)
  - Cumulative social risk plays a key role in life course epidemiology, including parenting and infant development, early childhood, and transition to adulthood
